# Efficacy of non-pharmacological interventions for alleviating insomnia in individuals with generalized anxiety disorder: systematic evaluation and net meta-analysis

**DOI:** 10.3389/fpsyt.2025.1669888

**Published:** 2025-10-31

**Authors:** Boyu Zhang, Yiwen Li, Wenming Chu, Yun Li, Jing Zhang, Zhenhao Lv, Ying Luo, Yan Chen

**Affiliations:** ^1^ Henan University of Chinese Medicine, Zhengzhou, China; ^2^ Third Affiliated Hospital of Henan University of Traditional Chinese Medicine, Zhengzhou, China; ^3^ Rehabilitation Medicine College, Henan University of Chinese Medicine, Zhengzhou, China

**Keywords:** non-pharmacological interventions, sleep, insomnia, anxiety, GAD, network meta-analysis

## Abstract

**Introduction:**

Anxiety is closely related to sleep, and the two often interact with each other. Generalized anxiety disorder (GAD) is often accompanied by insomnia, but pharmacologic interventions are typically ineffective and cause safety concerns. Despite the potential of non-pharmacological interventions (NIPs), their relative efficacy has not been clarified. The network meta-analysis (NMA) aims to explore the impact of NIPs on alleviating insomnia symptoms in patients with GAD.

**Methods and analysis:**

24 randomized controlled trials (1,953 patients) evaluating 14 NIPs for GAD-related insomnia were utilized in this NMA. This study systematically searched the databases including PubMed, Web of Science, EMBASE, Cochran, CNKI, SinoMed, VIP, and Wanfang. Meanwhile, Bayesian method was employed in conjunction with Markov Chain Monte Carlo (MCMC) simulation. In addition, this study conducted statistical analyses by using R (version 4.4.1) and STATA (version 15.1). Interventions were ranked by standardized mean difference (SMD) and surface under the cumulative ranking curve (SUCRA); study quality was evaluated using the Cochrane Risk of Bias tool (ROB2.0). Sleep quality was assessed with Pittsburgh Sleep Quality Index (PSQI) as well as Insomnia Severity Index (ISI), and anxiety symptoms were measured using Hamilton Anxiety Scale (HAMA) and Self-rating Anxiety Scale (SAS).

**Results:**

Acupuncture (AC) showed the best efficacy in improving sleep quality and alleviating anxiety symptoms. The combination of transcranial magnetic stimulation with psychotherapy (TMS+PT) significantly improved sleep quality and alleviating anxiety symptoms. Other interventions (e.g., relaxation therapy, exercise therapy, etc.) had limited efficacy.

**Conclusion:**

AC and TMS+PT are the best NIPs to improve insomnia and relieve anxiety in GAD patients. In the future, conducting multicenter trials and in-depth mechanistic studies is expected to validate the efficacy and optimize the individualized treatment regimen.

**Systematic Review Registration:**

https://www.crd.york.ac.uk/prospero/, identifier, CRD420251010334.

## 1 Introduction

As the most common subtype of anxiety disorders, generalized anxiety disorder (GAD) is prevalent in adults, with an incidence rate of 4.1% to 6.6% (1). It is primarily manifested by excessive worry, persistent tension, attention-deficit disorder, panic attacks, easy fatigue and sleep disorders and others (2). As people’s pace of life accelerates and social pressure increases, the prevalence and co-morbidity rates of GAD are increasing year by year, making it a prevalent global health problem. Insomnia represents one of the prevalent symptoms in patients with GAD and is a component of the diagnostic criteria for GAD, showing a complex and interactive relationship between the two (3). A survey (4) indicated that about 74% of GAD patients were found with insomnia symptoms during periods of anxiety. Another study (5) demonstrated that anxiety-specific interventions were ineffective when insomnia problems were not addressed. The superimposed clinical effect produced by anxiety and insomnia problems therefore suggests the need to further explore therapeutic approaches targeted at improving insomnia symptoms in GAD patients.

The conventional clinical approach to managing GAD patients with insomnia is the combination of anxiolytic and hypnotic sedative medications. However, 50% of patients exhibit suboptimal responses to anxiolytics (6), while long-term use of hypnotic sedative drugs may induce drug dependence as well as tolerance (7). Thus, there is a pressing need to explore safer and more efficacious treatments. Non-pharmacological interventions (NIPs) for sleep, which do not involve the use of medications, are characterized by low risk, low cost and significant efficacy, representing a promising technique for improving insomnia symptoms in patients with GAD (8).

Currently, NIPs to alleviate insomnia include psychotherapy (PT), transcranial magnetic stimulation (TMS), relaxation therapy (RT), exercise therapy (ET), acupuncture (AC), and tuina (TN) (9). PT comprises cognitive behavioral therapy (CBT), mindfulness therapy (MT), and sleep hygiene education. A meta-analysis revealed that CBT significantly enhanced sleep quality in psychiatric patients with comorbid insomnia symptoms, and the treatment remained effective after 18 months of follow-up (10). Another randomized controlled trial (RCT) study demonstrated that MT proved to be effective in improving sleep quality in patients with anxiety disorders (11). TMS, a promising technique for the treatment of psychiatric disorders, is effective for both anxiety and insomnia symptoms in GAD patients when they receive 1 Hz low-frequency TMS in the parietal cortex (12). RT assists patients in reducing somatic tension and related symptoms of depression and anxiety, thereby facilitating better sleep (13). Other NIPs, such as AC, TN, Baduanjin, Taiji, and yoga, can alleviate patients’ anxiety and insomnia symptoms, thereby providing important implications for improving patients’ outcomes and life quality (14–17).

Despite the positive effects of NIPs on sleep quality in GAD patients demonstrated in many studies, these investigations exclusively focus on a single intervention for improving sleep and lack direct or indirect comparisons across different interventions. Consequently, it is impossible to rank these NIPs based on their efficacy. Network meta-analysis (NMA), a statistical methodology, facilitates the synthesis of direct and indirect comparisons within a multi-therapy analytical framework and allows the assessment of the prioritization and ranking of multiple interventions (18). Therefore, this NMA was implemented to ascertain the efficacy of currently available NIPs in alleviating insomnia and anxiety symptoms in GAD patients, thus providing evidence-based support for the optimization of individualized therapeutic strategies in clinical practice.

## 2 Methods

This study adhered to the *Preferred Reporting Items for Systematic Evaluation and Meta-Analysis (PRISMA)* guidelines and its requirements for NMA (19). We have completed the PRISMA 2020 Checklist and the PRISMA 2020 for Abstract Checklist, available in **Supplementary Files S1** and **S2**, respectively. The study protocol has been registered in the PROSPERO (CRD420251010334). In accordance with the PRISMA 2020 statement, we report the following amendments made to the study protocol after registration. First, the target condition was refined from “anxiety disorders” to generalized anxiety disorder (GAD) to enhance clinical specificity, as insomnia represents one of the most prevalent symptoms in GAD patients, and the relationship between the two is complex and interactive. Second, the search strategy was expanded to include additional databases to improve retrieval comprehensiveness. All amendments were finalized prior to conducting the quantitative synthesis and did not alter the predetermined outcomes or eligibility criteria.

### 2.1 Search strategy

The publications in eight databases were searched in the study (PubMed, Web of Science, EMBASE, Cochran, CNKI, SinoMed, VIP, Wanfang). The time frame ranged from the construction of the database to March 11, 2025, and the language was confined to Chinese and English. The search was conducted employing a synthesis of subject terms and free words. The former one included Anxiety, Anxiety Disorder*, Insomnia, Sleep Quality, Sleep Initiation and Maintenance Disorders, RCT and Randomized Controlled Trial. Meanwhile, we manually searched for ClinicalTrials.gov (2020-2025) and WHO ICTRP platforms to screen for unpublished RCTS, and we did not identify unpublished studies. The specific search strategy used is described in **Supplementary File S3**.

### 2.2 Inclusion and exclusion criteria

According to the PICOS framework, literature conforming to the following criteria was included in this NMA: (i) P (Population): GAD patients with insomnia symptoms, where GAD meets the diagnostic criteria of the *Chinese Classification and Diagnostic Criteria Chinese Classification of Mental Disorders, Third Edition* (CCMD-III) (20), the *International Classification of Diseases, Eleventh Edition* (ICD-11) (21), or the Diagnostic and Statistical Manual of Mental Disorders, Fifth Edition (DSM-5) (3), and who are not comorbid with other psychiatric disorders. (ii) I (Intervention): NIPs were administered to at least one group of subjects. NIPs refer to therapeutic methods to achieve a therapeutic effect with no use of medications (22), including PT, TMS, RT, ET, AC, TN, and others. (iii)C (Comparison): Treatment as usual (TAU), placebo, sham surgery, waitlist, and other NIPs. Since placebo, sham surgery, and waitlist interventions showed no significant effect on the outcome measures of the subjects, the two authors considered combining them into a “Control group”. (iv) O (Outcome): The primary outcome indicator was sleep quality assessed by overall scores from self-rating sleep scales, encompassing the Pittsburgh Sleep Quality Index (PSQI) (23), Insomnia Severity Index (ISI) (24), PROMIS Sleep Disturbance short form (25), and Self-rating Scale of Sleep (SRSS) (26). Secondary outcome indicators referred to the severity of anxiety measured using self-rating measures such as the Hamilton Anxiety Scale (HAMA) (27), Self-rating Anxiety Scale (SAS) (28), Beck Anxiety Inventory (BAI) (29), and Generalized Anxiety Disorder 7 - item Scale (GAD-7) (30). (v) S (Study): Randomized controlled trial (RCT), with or without blinding.

Literature meeting the following criteria was excluded: (i) Those involving animal or cellular experiments, case reports, scientific experiment protocols, commentaries, letters, editorials, conference papers; (ii) Studies with missing data or significant errors; (iii) Duplicate publications; (iv) Unavailable full text; (v) Literature with duplicate participants included in the study; (vi) Insomnia induced by other psychiatric disorders, including depression, schizophrenia, post-traumatic stress disorder, or drug side effects; (vii) Patients taking sleeping pills or other sleep aids.

### 2.3 Study selection and data extraction

The retrieved documents were imported into EndNote 21.2. Two researchers (Yun Li and Jing Zhang) independently checked the titles and abstracts as per the inclusion and exclusion criteria, and then read the full texts. Subsequently, both researchers independently performed data extraction using Microsoft Excel 2023. The extracted data included: (a) basic information (first author, publication year, country, study design); (b) participant characteristics (sample size, sex ratio); (c) intervention and control measures (type, duration, follow-up period); and (d) outcomes and measurement tools. During both study selection and data extraction processes, the researchers remained blinded to each other’s entries. Any disagreements were resolved through consultation with a senior researcher (Yan Chen) for final adjudication.

### 2.4 Quality assessment

Two researchers (Zhenhao Lv and Yiwen Li) appraised the quality of eligible studies independently by applying the Cochrane Risk of Bias tool (ROB2.0) (31). The ROB2.0 criteria included five domains: generation of random sequences, allocation concealment, blinding, missing data, and selective reporting, each of which was rated as “high risk,” “some risk,” or “low risk”. For evaluating the overall quality of the articles, if all five domains were rated as low risk or only one domain was rated as medium risk with the rest being low risk, the overall risk of bias in the study was low; if four or more of the five domains were rated as medium risk or any one was rated as high risk, the overall risk of bias in the article was high; and the overall risk of bias in the study was medium for the rest of the scenarios. Two authors assessed the literature quality independently, and a third author (Yun Li) addressed dissents between the two researchers.

### 2.5 Statistical analysis

The included studies assessed outcomes with different scales, and the outcome indicators were continuous variables. Therefore, the standardized mean difference (SMD) was adopted as the effect size. In this study, a Markov Chain Monte Carlo (MCMC) method was leveraged to construct a Bayesian NMA model, with iterations of the model to evaluate the relative efficacy of various treatment regimens (18). The testing process was performed with a model chain value of 4, an annealing value of 10,000, an iteration value of 50,000, a step size of 10 for each test, and a baseline value of 2.5. This process aimed to get the posterior distribution (32). The NMA required three basic assumptions to be met, namely the assumption of transmissibility, homogeneity, and consistency. Heterogeneity analysis was carried out with the mtc:anohe function of the GeMTC package. Heterogeneity across included studies between the same comparisons was considered acceptable when the overall I^2^ was <50%, satisfying the homogeneity assumption. Inconsistency between direct and indirect comparisons was tested by node splitting using the mtc.nodesplit function in the GeMTC.package. When p > 0.05, it was considered that there was no inconsistency between direct and indirect comparisons, meeting the assumption of consistency (33). The consistency model was compared with the inconsistency model using the Deviance Information Criterion (DIC). A DIC difference of ≥5 was considered to indicate a significant improvement in model fit (suggesting potential inconsistency), whereas a smaller difference was interpreted as the absence of global inconsistency. The results were judged for convergence by computing the potential scale reduction factor (PSRF). With 1 as the standard, 1 ≤ PSRF < 1.05 was considered as successful convergence. A network structure was constructed, with the relationship of SMD between the interventions as lines and the interventions as nodes. Nodes denoted different interventions, and connecting lines indicated head-to-head comparisons across interventions. Cumulative probability ranking plots were leveraged to estimate all cumulative ranking probabilities and report them as the SUCRA, i.e., cumulative probability ranking; funnel plots and egger’s test were utilized to evaluate possible publication bias. All included studies had complete data with no missing values; therefore, no imputation or other missing-data procedures were required. All statistical analyses described above were performed using R (version 4.4.1) and STATA (version 15.1).

Among the included studies, one was a three-arm RCT comparing control group, scalp acupuncture and body acupuncture (34). Scalp acupuncture and Body acupuncture are both types of acupuncture; they are distinguished by differences in the selection of acupuncture points. This study divided the single three-arm RCT into two two-arm RCTs to preserve the two-by-two comparative analytic framework, i.e., this multi-group trial was divided into two distinct comparisons: (A) control group vs. scalp acupuncture and (B) control group vs. body acupuncture. This approach enabled consistency in the assumption of independent treatment effects across studies in the NMA (35).

## 3 Results

### 3.1 Literature search and screening process

**Figure 1:**
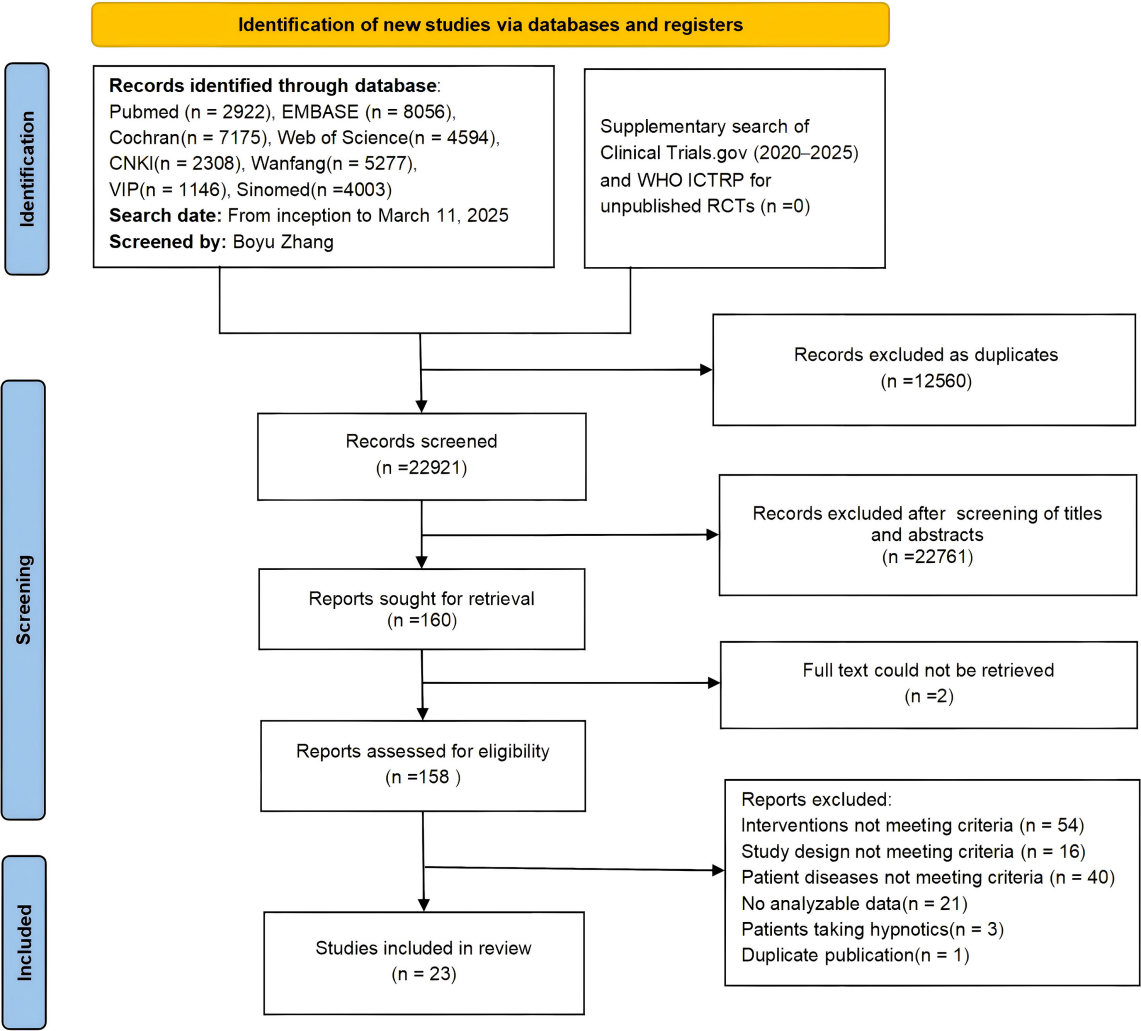
PRISMA 2020 flow diagram of literature screening process. Final included articles (n = 23) reporting on 24 two-arm RCTs (including 2 studies derived from one three-arm RCT).

A total of 35,481 records were searched from databases. After removing 12,560 duplicates, 22,761 records were excluded through preliminary screening of titles and abstracts. Two additional records were excluded because the full text could not be retrieved. We assessed 158 full-text articles for eligibility. Following strict application of the inclusion and exclusion criteria, 137 articles were excluded for not meeting the criteria, resulting in 23 articles being included in the final analysis. These 23 articles reported on 24 two-arm RCTs (including 2 studies derived from one three-arm RCT). The detailed screening process is shown in **Figure 1**.

### 3.2 Basic characteristics and quality assessment of included studies

Of the included studies, 22 were two-arm RCTs and one was a three-arm RCT; these trials were conducted in five countries: China, the United States, Germany, Sweden, and Iran. A total of 1953 patients were involved in the study, with a mean age of 40.55 years and 57.03% of female participants. A total of 14 interventions were included in the study, with a minimum intervention duration of 10 days and a maximum intervention duration of 12 weeks. Regarding the assessment of sleep quality, the PSQI was used in 19 studies, the ISI in 2 studies, the SRSS in 1 study, and the PROMIS Sleep Disturbance short form in 1 study. As for assessing anxiety symptoms, HAMA was used in ten studies, SAS in five studies, GAD-7 in three studies, and BAI in one study. The basic information about the included studies is specified in **Table 1**.

**Table 1:** Characteristics of included studies.

Author	Year	Countries	Design	Samplesize	Sex(M/F)	Intervention	Lasting days	Follow-up	Measurement (sleep)	Measurement (anxiety)
Guojuan Dong (34)	2018	China	RCT	18	21/39	Control group	4W	1W	PSQI	HAMA
				20		AC				
				20		AC				
Sang Na (36)	2023	China	RCT	60	30/30	PT	20D	No	PSQI	SAS
				60	30/30	PT + AC				
Fang Wang (15)	2022	China	RCT	36	30/42	RT	12W	No	PSQI	HAMA
				36		ET + RT				
Zhen Wang (37)	2023	China	RCT	43	20/23	RT	6W	No	PSQI	SAS
				43	21/22	SGB + RT				
Shan Zhao (38)	2024	China	RCT	41	19/22	PT	8W	No	PSQI	SAS
				42	23/19	TMS + PT				
Meng Yang (39)	2024	China	RCT	43	23/20	PT	8W	No	PSQI	SAS
				43	22/21	TMS + PT				
Chunfang Wang (40)	2021	China	RCT	51	27/24	TC	6W	No	SRSS	No
				51	29/22	PT + RT				
Dong Wang (41)	2021	China	RCT	44	16/28	TC	8W	No	ISI	SAS
				46	17/29	PT				
Wei Huang (42)	2024	China	RCT	30	7/23	Control group	2W	No	PSQI	HAMA
				30	8/22	TMS				
Junxia Gao (43)	2021	China	RCT	40	16/24	Control group	4W	No	PSQI	HAMA
				40	17/23	AC + RT				
Yan Huang (44)	2022	China	RCT	60	38/22	RT	8W	No	PSQI	HAMA
				60	35/25	AC + RT				
Junlin Mu (45)	2013	China	RCT	46	27/19	Control group	6W	No	PSQI	No
				46	29/17	RT				
Kun Liu (46)	2021	China	RCT	30	11/19	Control group	12W	No	PSQI	HAMA
				30	10/20	AC				
Yan Ma (17)	2016	China	RCT	30	10/20	Control group	10D	No	PSQI	HAMA
				30	11/19	AC + TN				
Yang Wang (47)	2019	China	RCT	35	18/17	Control group	6W	4W	PSQI	No
				36	17/19	PT				
Qilong Liu (48)	2020	China	RCT	40	15/25	Control group	No	No	PSQI	HAMA
				40	14/26	ET				
R. J. Jacoby (16)	2024	American	RCT	43	No	PT	12W	No	PSQI	No
				93		ET				
Lishu Gao (49)	2021	China	RCT	46	24/22	TMS	4W	No	PSQI	HAMA
				46	22/26	TMS + PT				
Siamak Khodarahimi (50)	2022	Iran	RCT	30	15/15	Control group	9W	12W	PSQI	GAD-7
				30	15/15	PT				
Johanna Boettcher (51)	2014	Germany	RCT	46	15/31	Control group	8W	24W	ISI	BAI
				45	11/34	PT				
Zhaoyang Huang (12)	2018	China	RCT	18	9/9	Control group	10D	4W	PSQI	HAMA
				18	9/9	TMS				
May Gao (11)	2022	American	RCT	35	3/32	Control group	8W	16W	PROMIS	GAD-7
				36	9/27	PT				
K. Jonsson (52)	2016	Sweden	RCT	25	8/17	Control group	7W	24W	PSQI	GAD-7
				25	7/18	RT				

AC, acupuncture; PT, Psychological therapy; RT, Relaxation therapy; ET, Exercise therapy; SGB, Stellate ganglion block; TMS, Transcranial Magnetic Stimulation; TC, Traditional care; M, male; F, female; W, week; D, day; No, Not Reported.

**Figure 2:**
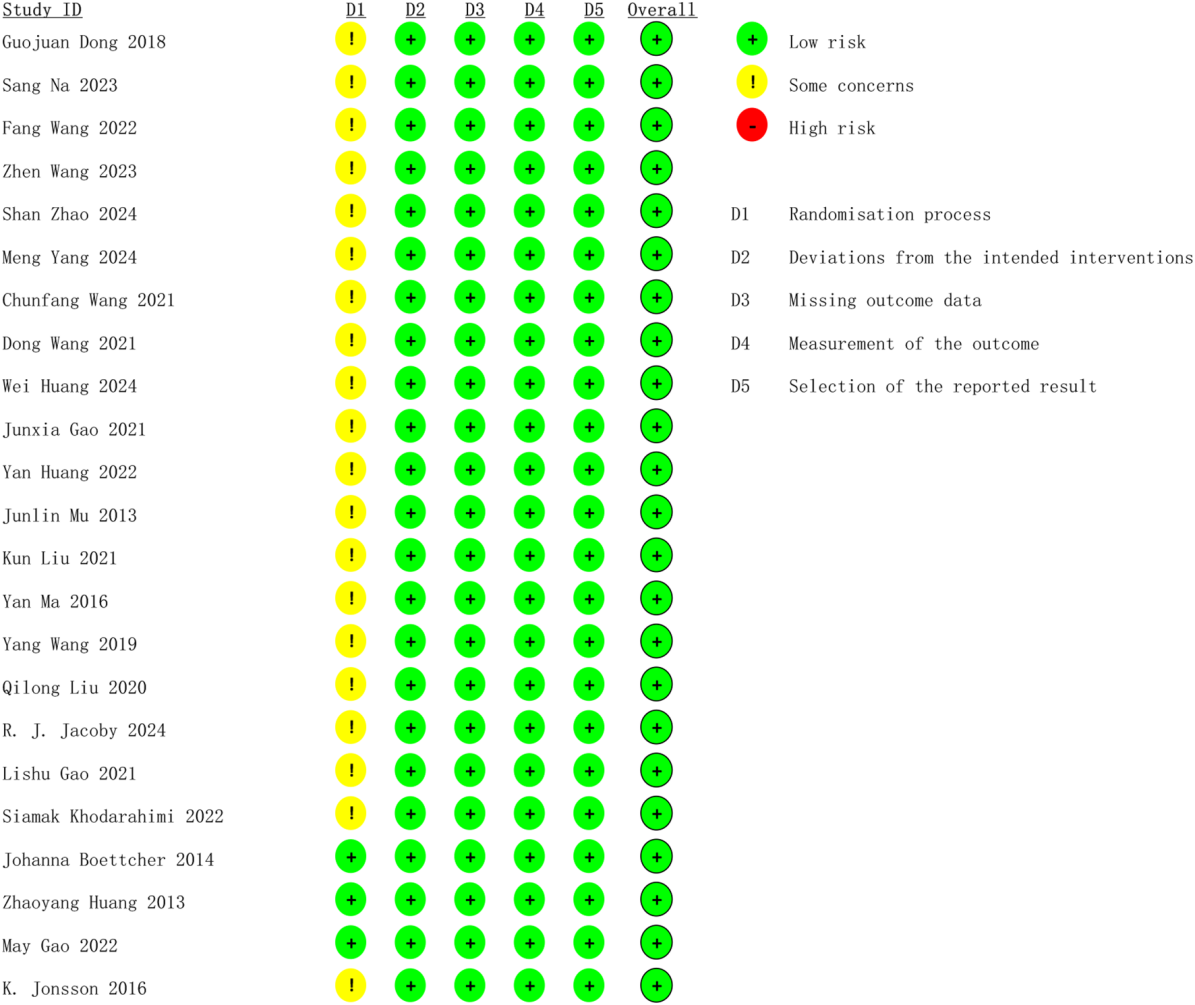
Risk of bias assessment for included studies using Cochrane ROB2.0 tool.

Concerning bias resulting from the random process, three studies were evaluated as low risk by clearly detailing how the randomized sequence was generated, how the group allocation was concealed, and the absence of baseline characteristics disparities between the two groups. The other 20 studies were classified as medium risk because of ambiguous randomization or inadequate allocation concealment. 23 studies were assessed as low risk for the other four possible aspects of bias. The overall bias risk of the included studies was assessed as low, suggesting that they were all high-quality studies. **Figure 2** displays the methodological quality assessment results of the included studies.

### 3.3 NMA results

#### 3.3.1 Network relationship diagram

**Figure 3:**
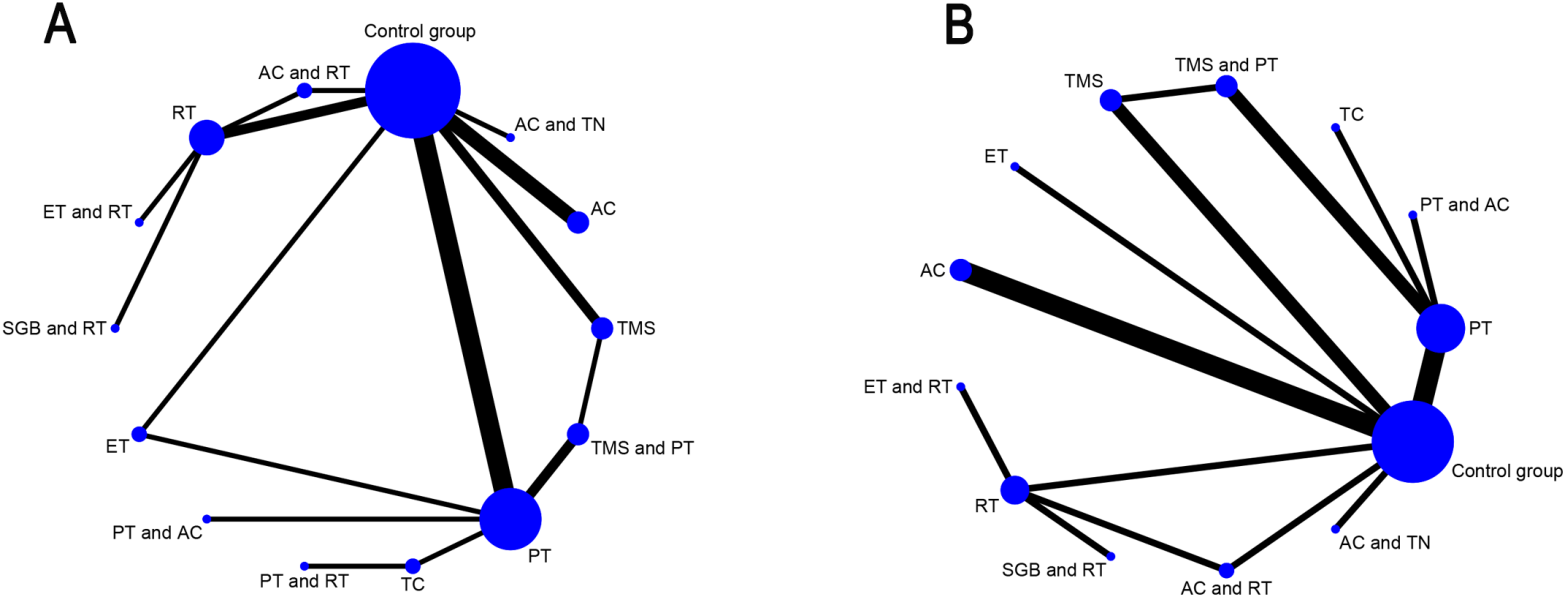
Network geometry of interventions for primary and secondary outcomes. **(A)** Sleep quality improvement in GAD patients; **(B)** Anxiety alleviation in GAD patients; AC, acupuncture; PT, Psychological therapy; RT, Relaxation therapy; ET, Exercise therapy; SGB, Stellate ganglion block; TMS, Transcranial Magnetic Stimulation; TC, Traditional care.

In the network relationship diagram, each dot expresses an intervention, and the dot size shows a positive correlation with the number of studies involved in each intervention, with larger dots indicating a greater number of studies included; The line connecting two dots signifies a direct comparative study between these two interventions, while the line thickness indicates the number of studies comparing two regimens, with a thicker line indicating a greater number of relevant comparative studies. **Figure 3** illustrate the detailed results. PSRF was calculated to determine the convergence of the results. The findings revealed that the PSRF values of all the outcome indicators were equal to 1 (**Supplementary File S4**), signifying complete convergence of the model. Node splitting was performed if there were closed loops. The results demonstrated that the P-values for all outcome indicators were greater than 0.05, indicating no local inconsistencies (**Supplementary File S5**). Furthermore, this study compared the fit of the consistency model with the inconsistency model using the Deviance Information Criterion (DIC). For the sleep scale, the DIC was 48.46361 for the consistency model and 48.18715 for the inconsistency model (difference < 5); for the anxiety scale, the DIC was 39.97305 for the consistency model and 39.86889 for the inconsistency model (difference < 5). As the DIC differences for both outcome measures were below the pre-specified threshold of 5, no significant global inconsistency was detected in the network.

#### 3.3.2 Primary outcome indicator: sleep quality

24 studies were included, including two two-arm RCTs (Guojuan Dong A and B) split from one three-arm RCT, and four self-rating sleep scales were utilized to assess post-intervention sleep quality. The scales used included the PSQI (23), ISI (24), PROMIS Sleep Disturbance short form (25), and SRSS (26). All scales were directionally consistent, where higher scores denoted poorer sleep quality.

**Figure 4:**
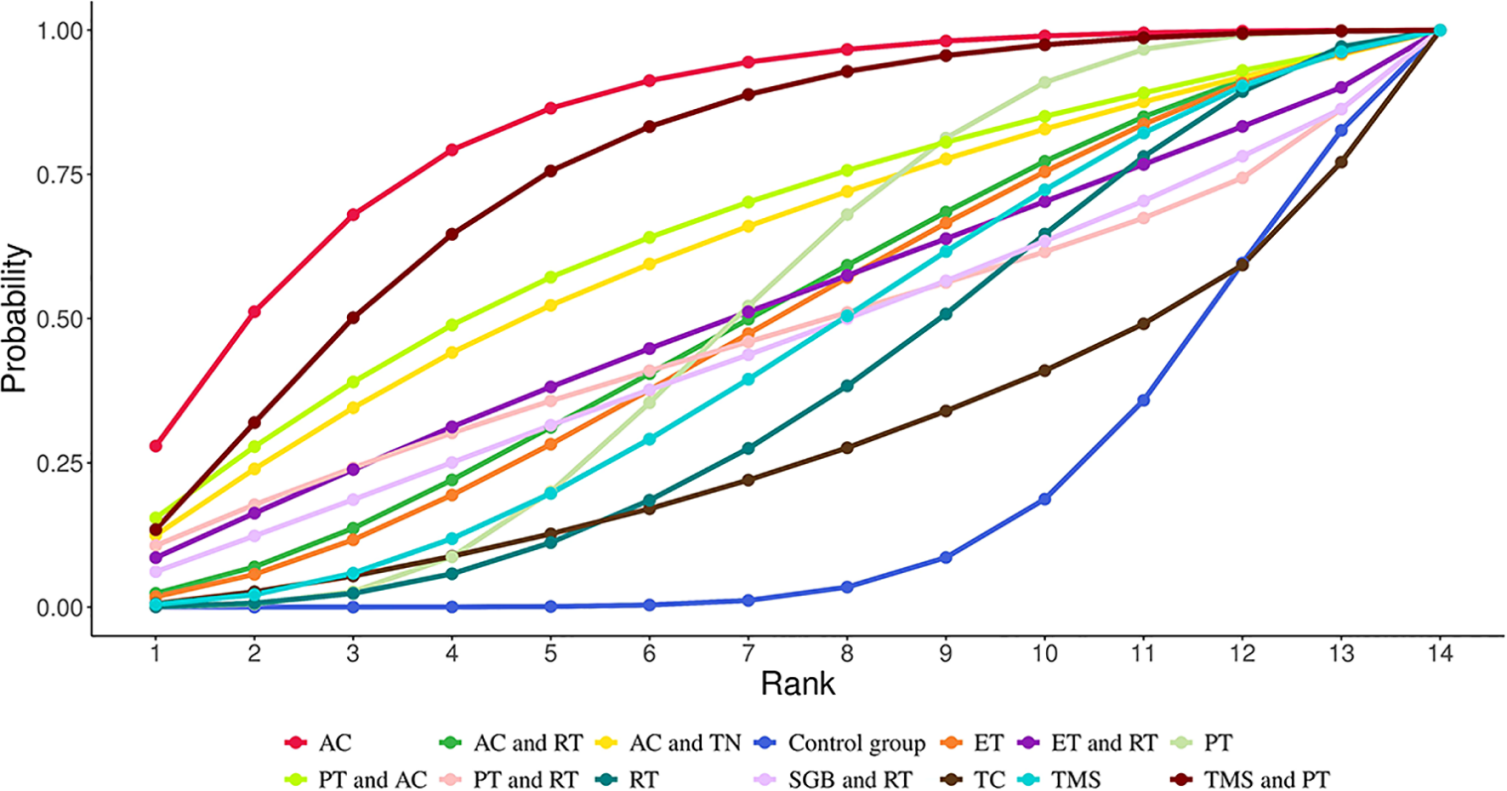
Cumulative Ranking Probabilities (SUCRA) of interventions for improving sleep quality in GAD patients. The Surface Under the Cumulative Ranking Curve (SUCRA) value represents the percentage of effectiveness, with 100% indicating the best intervention and 0% the worst.

NMA revealed that AC (SMD = 3.67, 95% CrI=1.34-6.09) and TMS+PT (SMD = 3.09, 95% CrI=0.32-5.89) significantly improved sleep quality in GAD patients compared with the control group, as shown in **Table 2**. The results of SUCRA probability ranking showed that AC (83.97%) > TMS and PT (76.29%) > PT and AC (64.80%) > AC and TN (61.59%). The greatest magnitude of improvement in patients’ sleep quality was achieved by AC alone, as shown in **Figure 4**.

**Table 2:** Head-to-head comparisons of efficacy and acceptability of non-pharmacological interventions for insomnia.

Control group	-3.67 (-6.09, -1.34)	-1.66 (-3.49, 0.1)	-2.59 (-6.99, 1.74)	-1.01 (-3.54, 1.52)	-1.67 (-6.37, 3.06)	-1.3 (-6, 3.41)	-3.09 (-5.89, -0.32)	-1.32 (-3.81, 1.17)	-1.61 (-4.69, 1.47)	-2.39 (-6.39, 1.63)	-1.53 (-4.52, 1.4)	-0.26 (-4.64, 4.09)	-1.34 (-7.25, 4.53)
3.67 (1.34, 6.09)	AC	2.01 (-0.97, 5.01)	1.08 (-3.92, 6.06)	2.65 (-0.76, 6.16)	2.01 (-3.23, 7.32)	2.36 (-2.87, 7.66)	0.58 (-3.05, 4.28)	2.36 (-1.05, 5.83)	2.06 (-1.79, 6.01)	1.29 (-3.34, 5.99)	2.14 (-1.63, 5.94)	3.42 (-1.52, 8.4)	2.32 (-4, 8.7)
1.66 (-0.1, 3.49)	-2.01 (-5.01, 0.97)	PT	-0.93 (-4.91, 3.07)	0.65 (-2.43, 3.79)	-0.01 (-5.01, 5.05)	0.36 (-4.64, 5.42)	-1.43 (-3.89, 1.09)	0.34 (-2.4, 3.15)	0.06 (-3.47, 3.66)	-0.72 (-5.06, 3.73)	0.13 (-2.81, 3.09)	1.41 (-2.58, 5.41)	0.32 (-5.3, 5.94)
2.59 (-1.74, 6.99)	-1.08 (-6.06, 3.92)	0.93 (-3.07, 4.91)	PT + AC	1.58 (-3.44, 6.65)	0.93 (-5.46, 7.39)	1.29 (-5.06, 7.72)	-0.5 (-5.19, 4.23)	1.27 (-3.56, 6.17)	0.99 (-4.37, 6.38)	0.2 (-5.65, 6.19)	1.06 (-3.88, 6.03)	2.34 (-3.31, 7.97)	1.25 (-5.64, 8.14)
1.01 (-1.52, 3.54)	-2.65 (-6.16, 0.76)	-0.65 (-3.79, 2.43)	-1.58 (-6.65, 3.44)	RT	-0.65 (-4.65, 3.32)	-0.29 (-4.25, 3.69)	-2.08 (-5.84, 1.69)	-0.3 (-3.88, 3.28)	-0.6 (-3.66, 2.49)	-1.36 (-6.1, 3.37)	-0.52 (-4.44, 3.34)	0.76 (-4.31, 5.77)	-0.33 (-6.77, 6.04)
1.67 (-3.06, 6.37)	-2.01 (-7.32, 3.23)	0.01 (-5.05, 5.01)	-0.93 (-7.39, 5.46)	0.65 (-3.32, 4.65)	ET + RT	0.37 (-5.28, 6.02)	-1.42 (-6.9, 4.05)	0.35 (-4.98, 5.68)	0.06 (-4.96, 5.12)	-0.71 (-6.9, 5.5)	0.13 (-5.45, 5.7)	1.41 (-5.07, 7.8)	0.33 (-7.27, 7.82)
1.3 (-3.41, 6)	-2.36 (-7.66, 2.87)	-0.36 (-5.42, 4.64)	-1.29 (-7.72, 5.06)	0.29 (-3.69, 4.25)	-0.37 (-6.02, 5.28)	SGB + RT	-1.79 (-7.29, 3.66)	-0.02 (-5.34, 5.32)	-0.31 (-5.32, 4.72)	-1.09 (-7.28, 5.13)	-0.24 (-5.82, 5.31)	1.04 (-5.42, 7.42)	-0.04 (-7.65, 7.45)
3.09 (0.32, 5.89)	-0.58 (-4.28, 3.05)	1.43 (-1.09, 3.89)	0.5 (-4.23, 5.19)	2.08 (-1.69, 5.84)	1.42 (-4.05, 6.9)	1.79 (-3.66, 7.29)	TMS + PT	1.77 (-1.16, 4.72)	1.49 (-2.65, 5.65)	0.7 (-4.15, 5.62)	1.55 (-2.2, 5.29)	2.84 (-1.88, 7.52)	1.74 (-4.4, 7.86)
1.32 (-1.17, 3.81)	-2.36 (-5.83, 1.05)	-0.34 (-3.15, 2.4)	-1.27 (-6.17, 3.56)	0.3 (-3.28, 3.88)	-0.35 (-5.68, 4.98)	0.02 (-5.32, 5.34)	-1.77 (-4.72, 1.16)	TMS	-0.3 (-4.23, 3.69)	-1.07 (-5.77, 3.68)	-0.21 (-3.98, 3.53)	1.07 (-3.83, 5.9)	-0.02 (-6.33, 6.2)
1.61 (-1.47, 4.69)	-2.06 (-6.01, 1.79)	-0.06 (-3.66, 3.47)	-0.99 (-6.38, 4.37)	0.6 (-2.49, 3.66)	-0.06 (-5.12, 4.96)	0.31 (-4.72, 5.32)	-1.49 (-5.65, 2.65)	0.3 (-3.69, 4.23)	AC + RT	-0.77 (-5.81, 4.3)	0.07 (-4.23, 4.32)	1.35 (-4.03, 6.68)	0.26 (-6.41, 6.92)
2.39 (-1.63, 6.39)	-1.29 (-5.99, 3.34)	0.72 (-3.73, 5.06)	-0.2 (-6.19, 5.65)	1.36 (-3.37, 6.1)	0.71 (-5.5, 6.9)	1.09 (-5.13, 7.28)	-0.7 (-5.62, 4.15)	1.07 (-3.68, 5.77)	0.77 (-4.3, 5.81)	AC + TN	0.85 (-4.16, 5.81)	2.13 (-3.84, 8.02)	1.05 (-6.17, 8.14)
1.53 (-1.4, 4.52)	-2.14 (-5.94, 1.63)	-0.13 (-3.09, 2.81)	-1.06 (-6.03, 3.88)	0.52 (-3.34, 4.44)	-0.13 (-5.7, 5.45)	0.24 (-5.31, 5.82)	-1.55 (-5.29, 2.2)	0.21 (-3.53, 3.98)	-0.07 (-4.32, 4.23)	-0.85 (-5.81, 4.16)	ET	1.28 (-3.69, 6.25)	0.19 (-6.16, 6.51)
0.26 (-4.09, 4.64)	-3.42 (-8.4, 1.52)	-1.41 (-5.41, 2.58)	-2.34 (-7.97, 3.31)	-0.76 (-5.77, 4.31)	-1.41 (-7.8, 5.07)	-1.04 (-7.42, 5.42)	-2.84 (-7.52, 1.88)	-1.07 (-5.9, 3.83)	-1.35 (-6.68, 4.03)	-2.13 (-8.02, 3.84)	-1.28 (-6.25, 3.69)	TC	-1.09 (-5.07, 2.88)
1.34 (-4.53, 7.25)	-2.32 (-8.7, 4)	-0.32 (-5.94, 5.3)	-1.25 (-8.14, 5.64)	0.33 (-6.04, 6.77)	-0.33 (-7.82, 7.27)	0.04 (-7.45, 7.65)	-1.74 (-7.86, 4.4)	0.02 (-6.2, 6.33)	-0.26 (-6.92, 6.41)	-1.05 (-8.14, 6.17)	-0.19 (-6.51, 6.16)	1.09 (-2.88, 5.07)	TC

Negative SMD values indicate the intervention group is superior to the control group.

#### 3.3.3 Secondary outcome indicator: anxiety level

20 studies were included, including two two-arm studies (Guojuan Dong A and B) split from one three-arm studies, and four self-rating anxiety scales were utilized to assess post-intervention anxiety. The scales used included the HAMA (27), SAS (28), BAI (29), and GAD-7 (30). All scales were directionally consistent, where higher scores denoted more severe anxiety.

**Figure 5:**
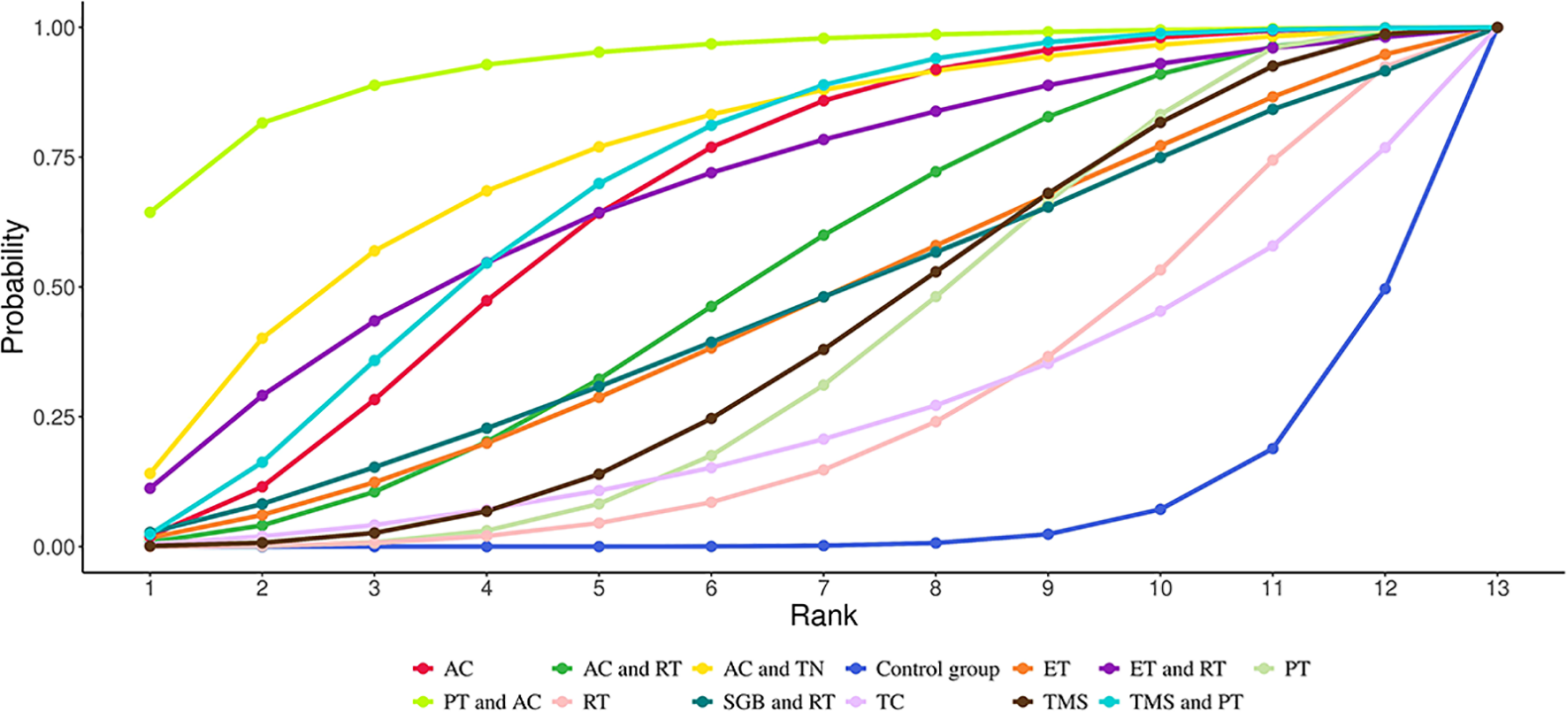
Cumulative Ranking Probabilities (SUCRA) of interventions for alleviating anxiety in GAD patients. The Surface Under the Cumulative Ranking Curve (SUCRA) value represents the percentage of effectiveness, with 100% indicating the best intervention and 0% the worst.

NMA showed that compared with the control group, AC (SMD = 2.20, 95% CrI=0.90-3.50), PT (SMD = 1.24, 95% CrI=0.10-1.24), AC+TN (SMD = 2.71, 95% CrI=0.48- 4.98), PT+AC (SMD = 3.91, 95% CrI=1.45-6.45), TMS+PT (SMD = 2.30, 95% CrI=0.72-3.94) significantly relieved anxiety of GAD patients, as illustrated in **Table 3**. The results of SUCRA probability ranking revealed that PT and AC (92.88%) > AC and TN (75.69%) > TMS and PT (69.87%) > AC (66.76%). PT+AC was the most effective for ameliorating patients’ anxiety, as shown in **Figure 5**.

**Table 3:** Head-to-head comparisons of efficacy and acceptability of non-pharmacological interventions for anxiety.

Control group	-2.2 (-3.5, -0.9)	-1.24 (-2.46, -0.1)	-3.91 (-6.45, -1.45)	-0.82 (-2.63, 0.99)	-2.36 (-5.2, 0.48)	-1.43 (-4.26, 1.42)	-2.3 (-3.94, -0.72)	-1.31 (-2.7, 0.07)	-1.66 (-3.45, 0.13)	-2.71 (-4.98, -0.48)	-1.45 (-3.64, 0.76)	-0.64 (-3.18, 1.8)
2.2 (0.9, 3.5)	AC	0.96 (-0.83, 2.67)	-1.71 (-4.55, 1.06)	1.37 (-0.85, 3.61)	-0.16 (-3.27, 2.98)	0.77 (-2.35, 3.88)	-0.1 (-2.19, 1.93)	0.89 (-1.02, 2.79)	0.54 (-1.67, 2.76)	-0.51 (-3.14, 2.07)	0.75 (-1.79, 3.32)	1.56 (-1.3, 4.32)
1.24 (0.1, 2.46)	-0.96 (-2.67, 0.83)	PT	-2.67 (-4.87, -0.48)	0.41 (-1.7, 2.62)	-1.12 (-4.15, 1.99)	-0.19 (-3.23, 2.93)	-1.06 (-2.42, 0.31)	-0.07 (-1.65, 1.58)	-0.42 (-2.53, 1.77)	-1.48 (-3.99, 1.1)	-0.21 (-2.65, 2.34)	0.59 (-1.61, 2.79)
3.91 (1.45, 6.45)	1.71 (-1.06, 4.55)	2.67 (0.48, 4.87)	PT + AC	3.08 (0.06, 6.22)	1.55 (-2.18, 5.37)	2.48 (-1.27, 6.3)	1.61 (-0.98, 4.2)	2.6 (-0.09, 5.37)	2.25 (-0.77, 5.37)	1.19 (-2.13, 4.57)	2.46 (-0.8, 5.84)	3.26 (0.18, 6.38)
0.82 (-0.99, 2.63)	-1.37 (-3.61, 0.85)	-0.41 (-2.62, 1.7)	-3.08 (-6.22, -0.06)	RT	-1.54 (-3.73, 0.66)	-0.61 (-2.79, 1.58)	-1.48 (-3.94, 0.91)	-0.48 (-2.77, 1.79)	-0.83 (-2.64, 0.94)	-1.88 (-4.8, 0.99)	-0.62 (-3.47, 2.22)	0.18 (-2.93, 3.23)
2.36 (-0.48, 5.2)	0.16 (-2.98, 3.27)	1.12 (-1.99, 4.15)	-1.55 (-5.37, 2.18)	1.54 (-0.66, 3.73)	ET + RT	0.93 (-2.18, 4.02)	0.05 (-3.25, 3.29)	1.05 (-2.12, 4.2)	0.71 (-2.16, 3.52)	-0.35 (-4.01, 3.26)	0.91 (-2.67, 4.51)	1.72 (-2.1, 5.44)
1.43 (-1.42, 4.26)	-0.77 (-3.88, 2.35)	0.19 (-2.93, 3.23)	-2.48 (-6.3, 1.27)	0.61 (-1.58, 2.79)	-0.93 (-4.02, 2.18)	SGB + RT	-0.87 (-4.17, 2.35)	0.12 (-3.04, 3.28)	-0.23 (-3.06, 2.59)	-1.28 (-4.92, 2.34)	-0.01 (-3.61, 3.59)	0.79 (-3.04, 4.52)
2.3 (0.72, 3.94)	0.1 (-1.93, 2.19)	1.06 (-0.31, 2.42)	-1.61 (-4.2, 0.98)	1.48 (-0.91, 3.94)	-0.05 (-3.29, 3.25)	0.87 (-2.35, 4.17)	TMS + PT	0.99 (-0.64, 2.68)	0.64 (-1.74, 3.06)	-0.41 (-3.15, 2.37)	0.85 (-1.84, 3.61)	1.66 (-0.94, 4.22)
1.31 (-0.07, 2.7)	-0.89 (-2.79, 1.02)	0.07 (-1.58, 1.65)	-2.6 (-5.37, 0.09)	0.48 (-1.79, 2.77)	-1.05 (-4.2, 2.12)	-0.12 (-3.28, 3.04)	-0.99 (-2.68, 0.64)	TMS	-0.35 (-2.62, 1.92)	-1.41 (-4.05, 1.23)	-0.14 (-2.72, 2.49)	0.66 (-2.09, 3.35)
1.66 (-0.13, 3.45)	-0.54 (-2.76, 1.67)	0.42 (-1.77, 2.53)	-2.25 (-5.37, 0.77)	0.83 (-0.94, 2.64)	-0.71 (-3.52, 2.16)	0.23 (-2.59, 3.06)	-0.64 (-3.06, 1.74)	0.35 (-1.92, 2.62)	AC + RT	-1.05 (-3.94, 1.81)	0.21 (-2.6, 3.04)	1.01 (-2.09, 4.06)
2.71 (0.48, 4.98)	0.51 (-2.07, 3.14)	1.48 (-1.1, 3.99)	-1.19 (-4.57, 2.13)	1.88 (-0.99, 4.8)	0.35 (-3.26, 4.01)	1.28 (-2.34, 4.92)	0.41 (-2.37, 3.15)	1.41 (-1.23, 4.05)	1.05 (-1.81, 3.94)	AC + TN	1.26 (-1.86, 4.41)	2.07 (-1.32, 5.4)
1.45 (-0.76, 3.64)	-0.75 (-3.32, 1.79)	0.21 (-2.34, 2.65)	-2.46 (-5.84, 0.8)	0.62 (-2.22, 3.47)	-0.91 (-4.51, 2.67)	0.01 (-3.59, 3.61)	-0.85 (-3.61, 1.84)	0.14 (-2.49, 2.72)	-0.21 (-3.04, 2.6)	-1.26 (-4.41, 1.86)	ET	0.81 (-2.58, 4.07)
0.64 (-1.8, 3.18)	-1.56 (-4.32, 1.3)	-0.59 (-2.79, 1.61)	-3.26 (-6.38, -0.18)	-0.18 (-3.23, 2.93)	-1.72 (-5.44, 2.1)	-0.79 (-4.52, 3.04)	-1.66 (-4.22, 0.94)	-0.66 (-3.35, 2.09)	-1.01 (-4.06, 2.09)	-2.07 (-5.4, 1.32)	-0.81 (-4.07, 2.58)	TC

Negative SMD values indicate the intervention group is superior to the control group.

### 3.4 Publication bias

**Figure 6:**
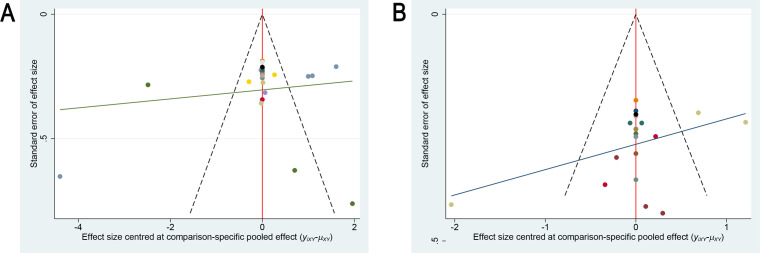
Comparison-adjusted funnel plots for publication bias assessment. **(A)** Sleep quality outcome; **(B)** Anxiety outcome.

A correction-comparison funnel plot was utilized to test publication bias. The funnel plots were symmetrical, suggesting no publication bias, as shown in **Figure 6**. The results of egger’s test indicated no significant publication bias for both the primary outcome (sleep quality, P = 0.549) and the secondary outcome (anxiety severity, P = 0.467) (all P > 0.05), which is consistent with the funnel plot findings.

## 4 Discussion

### 4.1 Summary

In NMA, 24 two-arm RCTs evaluating the efficacy of NIPs to improve insomnia symptoms in GAD patients were included, involving 14 interventions and 1,953 GAD patients with insomnia symptoms. Our meta-analysis demonstrated that compared to the control group, AC and TMS+PT significantly improved patients’ sleep quality; AC, PT, AC+PT, AC+TN, and TMS+PT significantly relieved patients’ anxiety. All differences were statistically significant. Results of the integrated evaluation of two outcome indicators indicated that AC and TMS+PT were the most effective interventions for GAD patients with insomnia symptoms.

### 4.2 Integrity and applicability of evidence

AC and TMS+PT have demonstrated significant superiority in improving insomnia symptoms in GAD patients.

AC has emerged as a promising technique, and a growing number of patients are strongly requesting AC therapies to treat sleep disorders (53, 54). AC refers to the practice of inserting sterile tiny needles or using infrared light generated by moxibustion to stimulate specific areas of human skin under the guidance of TCM theory. These areas are called acupoints, modulating qi and blood and harmonizing yin and yang. AC is safe, user-friendly, and tolerable with no side effects (55). A meta-analysis showed (56) that AC performed remarkably well in improving patients’ psychological symptoms, especially insomnia and anxiety, with significant reductions in PSQI, HAMD, and SAS, and mild adverse effects, compared with TAU or conventional medications. Another meta-analysis demonstrated (57) that AC presented a therapeutic advantage over benzodiazepines and sham AC in treating insomnia and anxiety in patients. Nevertheless, GAD patients were not the target of these studies. The evidence for AC for improving insomnia symptoms in GAD patients remains absent. This study employed NMA to ascertain the efficacy of 14 NIPs for insomnia in GAD patients, filling a knowledge gap in this area. It was found that AC exerted a significant superiority in ameliorating both insomnia symptoms and anxiety levels in GAD patients compared to the control group. Laboratory studies confirmed that AC, by modulating HPA axis activity (58), regulating SNS function (59), balancing circadian rhythms, and influencing the cytokines (e.g., IL-1, IL-6) and neurotransmitters (e.g., neuropeptide Y, 5-hydroxytryptophan, melatonin, dopamine, norepinephrine, γ-aminobutyric acid, and β-endorphin) (60–62), was beneficial in improving sleep quality and anxiety levels in patients. These targets highly matched the molecular mechanisms underlying the treatment of psychiatric disorders such as anxiety disorders (63). In terms of combination therapy, previous studies have mostly focused on CBT + medication, while the evidence for the effectiveness of non-pharmacological multimodal regimens is lacking. SUCRA values for PT+AC (64.80%) were found to be significantly higher than those for PT alone (50.43%), while RT+AC also had higher SUCRA values (49.55%) compared to RT alone (37.26%). This result aligned with the results of a prior meta-analysis (64), suggesting that AC may enhance the efficacy of NIPs through multi-targeted modulation.

TMS is a technique for modulating cerebral cortex activity through single or repetitive pulsed magnetic fields (65). TMS specifically induces sleep oscillations including slow waves and sleep spindle waves (65). For example, applying a single pulse of TMS at less than 1 Hz during non-rapid eye movement (NREM) sleep enhances slow wave activity (SWA), which is critical for brain function recovery. Due to the potential association of anxiety and related disorders with high reactivity to emotional stimuli (66), SWA-enriched sleep may function therapeutically by modulating such reactivity. In this study, TMS was not significantly different compared to control group and TAU. That is, TMS failed to significantly improve the sleep quality of GAD patients. This differs from the earlier review study results (67). Although clinical trials for GAD patients have demonstrated the potential of TMS in alleviating insomnia and anxiety symptoms, the effects of TMS need to be further corroborated by high-quality evidence. Notably, in terms of combination therapy, it was found that TMS+PT remarkably enhanced the sleep quality and anxiety of GAD patients in comparison with the control group, with significant differences.

Current PT for GAD is comprised of CBT, MT, and sleep hygiene education. CBT is the most well-researched and commonly used among them. PT eliminates or relieves patients’ fear of disease to a certain extent by enabling them to correctly understand the disease, further improving their confidence in treatment. Upon this basis, psychotherapists assist the patients in adjusting their work and rest, cultivating their concentration, sleep habits and so forth, enabling them to be more focused on falling asleep and integrating their fragmented light sleep time for the purpose of improving the patients’ sleep quality. Compared with the control group in this study, PT failed to exhibit a significant advantage in ameliorating sleep quality, though it significantly relieved anxiety in GAD patients. This result was in line with previous reviews. A meta-analysis evaluating the efficacy of MT for sleep disorders in adults demonstrated (68) that MT demonstrated no significant effect on ameliorating sleep quality. Another meta-analysis analyzing the influence of CBT on sleep disorders accompanying anxiety disorders also suggested (69) that CBT treatment for anxiety disorders provided only a moderate effect on improving insomnia symptoms (aggregated effect size of 0.527). Interestingly, this study found that neither PT nor TMS used alone was advantageous in improving sleep quality in GAD patients (both SUCRA values <50%). Nonetheless, the therapeutic effect is significantly enhanced when the two are applied in combination. TMS+PT demonstrated a remarkable advantage in improving both insomnia symptoms (SUCRA = 76.29%) and anxiety (SUCRA = 69.87%) second only to AC (SUCRA = 83.97%), with statistically significant effect sizes (SMD = 3.67, 95% CrI=1.34-6.09). This result challenges the conventional monotherapy-dominated therapeutic paradigm, indicating that combined interventions may address the complexity of GAD pathology through multi-target synergistic mechanisms. The synergistic effect of TMS and PT supports the scientific validity of “neuro-behavioral” integrated interventions, which provides a highly evidence-based strategy for the treatment of GAD comorbid insomnia. Future studies should further elucidate the molecular-loop mechanism and promote the clinical translation of the precise combination regimen.

### 4.3 Strengths and limitations

In terms of strengths, this study assessed for the first time the effectiveness of 14 NIPs on insomnia and anxiety symptoms in GAD patients through a Bayesian NMA, thereby addressing the lack of direct comparative evidence in this area. The MCMC algorithm was used to iteratively optimize the model and verify the convergence (PSRF = 1) and consistency (P>0.05 for node-splitting method) of the results, remarkably improving the robustness of the analysis. Furthermore, this study pioneers the prioritization of AC and combination therapies (such as TMS+PT) in improving insomnia symptoms in GAD patients (SUCRA >75%), providing a high-level evidence-based basis for clinical development of individualized treatment strategies.

However, this NMA retains several limitations that need to be identified. First, the type, composition, scoring criteria, and sensitivity of the insomnia and anxiety scales used in the included studies varied, potentially increasing between-study heterogeneity although SMD was applied as the effect size. Second, despite the acceptable overall heterogeneity of the studies (I² = 7%) under the random-effects model, the included studies differed significantly in the form of the intervention (e.g., types of Acupuncture, acupoint selection, TMS stimulation parameters, psychotherapeutic modalities) and session settings (10 days to 12 weeks), which may affect the incorporation and interpretation of effect sizes. Third, although this study identified several effective combined interventions, their specific protocols, such as the sequencing, duration, and integration of components, varied across the included trials. This heterogeneity limits our ability to formulate standardized clinical protocols. Fourth, as the included studies were dominated by Chinese populations, the efficacy of AC therapy, which has a rich cultural history and professional resources in China, may be affected by differences in cultural identity and operational standardization. Therefore, cross-cultural validation in Western populations is needed to clarify the generalizability of its efficacy. Finally, this NMA included a relatively limited number of studies, with some interventions represented in few papers, making these interventions under-researched. Finally, this study did not include several emerging non-pharmacological neuromodulation interventions, such as transcutaneous auricular vagus nerve stimulation (taVNS), due to reasons such as falling outside the predefined PICOS criteria or being published after the search cutoff date. A recent meta-analysis indicates that taVNS can significantly improve insomnia scores (70), however, the overall GRADE evidence quality remains very low, suggesting its potential while underscoring the need for higher-quality trials with longer-term follow-up. Subsequent research should consider incorporating interventions like taVNS into network meta-analyses.

### 4.4 Conclusion

This network meta-analysis showed that acupuncture (AC) and transcranial magnetic stimulation combined with psychotherapy (TMS+PT) produced the most pronounced improvements in sleep, PT+AC ranked among the top for reducing anxiety, and TMS+PT performed consistently across both outcomes, with statistically significant effect sizes. Notably, a considerable proportion of trials received RoB 2 judgments of “some concerns” for the randomization process, and long-term outcomes were infrequently reported, which lowers the certainty of the evidence. Future research should undertake larger, multicenter randomized controlled trials with rigorous allocation concealment and blinding where feasible, standardized intervention protocols and preregistered analyses, ≥6-month follow-up, and comprehensive safety reporting to evaluate the durability of benefits.

## Data Availability

The raw data supporting the conclusions of this article will be made available by the authors, without undue reservation.

## Supplementary material

Supplementary File S1 PRISMA 2020 Checklist.

Supplementary File S2 PRISMA 2020 for Abstract Checklist.

Supplementary File S3 Comprehensive Search Strategies for All Databases.

Supplementary File S4 Potential Scale Reduction Factor (PSRF) Plots for Convergence Diagnosis **(A)** Sleep quality outcome **(B)** Anxiety outcome.

Supplementary File S5 Node-Splitting Analysis for Local Inconsistency Assessment **(A, B)** Sleep quality outcome **(C, D)** Anxiety outcome.
