# Supplementary material for: Efficacy of non-pharmacological interventions for alleviating insomnia in individuals with generalized anxiety disorder: systematic evaluation and net meta-analysis
Source: Front Psychiatry. 2025 Oct 31;16:1669888. doi: 10.3389/fpsyt.2025.1669888 (PMC12615457; doi:10.3389/fpsyt.2025.1669888)
Supplement: Supplementary File S3 — Comprehensive Search Strategies for All Databases. [file SupplementaryFile3.pdf]

Table S1. The search strategy (Pubmed)

Search number	Query	Results
1	(Anxiety Disorders[MeSH Terms]) OR (Anxiety[MeSH Terms])	204,280
2	"Angst"[Title/Abstract] OR "Anxiety"[Title/Abstract] OR "Anxiety Disorder*"[Title/Abstract] OR "Anxiety Neuroses"[Title/Abstract] OR "Anxiousness"[Title/Abstract] OR "Hypervigilance"[Title/Abstract] OR "Nervousness"[Title/Abstract] OR "Neurotic Anxiety State*"[Title/Abstract] OR "Social Anxieties"[Title/Abstract] OR "Social Anxiety"[Title/Abstract]	307069
3	(Sleep Initiation and Maintenance Disorders[MeSH Terms]) OR (Sleep Quality[MeSH Terms])	22911
4	"agrypnia"[Title/Abstract] OR "Chronic Insomnia"[Title/Abstract] OR "DIMS"[Title/Abstract] OR "disorder of sleep initiation and maintenance"[Title/Abstract] OR "Disorders of Initiating and Maintaining Sleep"[Title/Abstract] OR "disorders of sleep initiation and maintenance"[Title/Abstract] OR "Early Awakening"[Title/Abstract] OR "hyposomnia"[Title/Abstract] OR "hyposomnias"[Title/Abstract] OR "Insomnia Disorder*"[Title/Abstract] OR "Insomnia*"[Title/Abstract] OR "Nonorganic Insomnia"[Title/Abstract] OR "Primary Insomnia"[Title/Abstract] OR "Psychophysiological Insomnia"[Title/Abstract] OR "Rebound Insomnia"[Title/Abstract] OR "Secondary Insomnia"[Title/Abstract] OR "sleep initiation and maintenance disorder"[Title/Abstract] OR "sleep initiation and maintenance disorders"[Title/Abstract] OR "Sleep Initiation Dysfunction"[Title/Abstract] OR "Sleep Initiation Dysfunctions"[Title/Abstract] OR "sleep initiation/maintenance disorder"[Title/Abstract] OR "Sleep Qualities"[Title/Abstract] OR "Sleep Quality"[Title/Abstract] OR "sleeplessness"[Title/Abstract] OR "Transient Insomnia"[Title/Abstract]	61955
5	"randomised controlled study"[Title/Abstract] OR "randomised controlled trial"[Title/Abstract] OR "randomized controlled study"[Title/Abstract] OR "randomized controlled trial"[Title/Abstract] OR "random*"[Title/Abstract] OR "trial, randomized controlled"[Title/Abstract] OR "Clinical Trials, Randomized"[Title/Abstract] OR "Trials, Randomized Clinical"[Title/Abstract]	1610123
6	(#1 OR #2) AND (#3 OR #4) AND #5	2922

Table S2. The search strategy (EMBASE)

Search number	Query	Results
1	'anxiety disorder'/exp OR 'anxiety'/exp	653675
2	'insomnia'/exp OR 'sleep quality'/exp	139369
3	'angst':ab,ti,kw OR 'anxiety':ab,ti,kw OR 'anxiety disorder*':ab,ti,kw OR 'anxiety neuroses':ab,ti,kw OR 'anxiousness':ab,ti,kw OR 'hypervigilance':ab,ti,kw OR 'nervousness':ab,ti,kw OR 'neurotic anxiety state*':ab,ti,kw OR 'social anxieties':ab,ti,kw OR 'social anxiety':ab,ti,kw	438412
4	'agrypnia':ab,ti,kw OR 'chronic insomnia':ab,ti,kw OR 'dims':ab,ti,kw OR 'disorder of sleep initiation and maintenance':ab,ti,kw OR 'disorders of initiating and maintaining sleep':ab,ti,kw OR 'disorders of sleep initiation and maintenance':ab,ti,kw OR 'early awakening':ab,ti,kw OR 'hyposomnia':ab,ti,kw OR 'hyposomnias':ab,ti,kw OR 'insomnia disorder*':ab,ti,kw OR 'insomnia*':ab,ti,kw OR 'nonorganic insomnia':ab,ti,kw OR 'primary insomnia':ab,ti,kw OR 'psychophysiological insomnia':ab,ti,kw OR 'rebound insomnia':ab,ti,kw OR 'secondary insomnia':ab,ti,kw OR 'sleep initiation and maintenance disorder':ab,ti,kw OR 'sleep initiation and maintenance disorders':ab,ti,kw OR 'sleep initiation dysfunction':ab,ti,kw OR 'sleep initiation dysfunctions':ab,ti,kw OR 'sleep initiation/maintenance disorder':ab,ti,kw OR 'sleep qualities':ab,ti,kw OR 'sleep quality':ab,ti,kw OR 'sleeplessness':ab,ti,kw OR 'transient insomnia':ab,ti,kw	99367
5	'randomised controlled study':ab,ti,kw OR 'randomised controlled trial':ab,ti,kw OR 'randomized controlled study':ab,ti,kw OR 'randomized controlled trial':ab,ti,kw OR 'random*':ab,ti,kw OR 'trial, randomized controlled':ab,ti,kw OR 'clinical trials, randomized':ab,ti,kw OR 'trials, randomized clinical':ab,ti,kw	2197473
6	(#1 OR #3) AND (#2 OR #4) AND #5	8056

Table S3. The search strategy (Cochran)

Search number	Query	Results
1	MeSH descriptor: [Anxiety Disorders] explode all trees	10493
2	MeSH descriptor: [Anxiety] explode all trees	13026
3	('Angst' OR 'Anxiety' OR 'Anxiety Disorder*' OR 'Anxiety Neuroses' OR 'Anxiousness' OR 'Hypervigilance' OR 'Nervousness' OR 'Neurotic Anxiety State*' OR 'Social Anxieties' OR 'Social Anxiety'):ab,ti,kw	84560
4	MeSH descriptor: [Sleep Initiation and Maintenance Disorders] explode all trees	3895
5	MeSH descriptor: [Sleep Quality] explode all trees	512
6	('agrypnia' OR 'Chronic Insomnia' OR 'DIMS' OR 'disorder of sleep initiation and maintenance' OR 'Disorders of Initiating and Maintaining Sleep' OR 'disorders of sleep initiation and maintenance' OR 'Early Awakening' OR 'hyposomnia' OR 'hyposomnias' OR 'Insomnia Disorder*' OR 'Insomnia*' OR 'Nonorganic Insomnia' OR 'Primary Insomnia' OR 'Psychophysiological Insomnia' OR 'Rebound Insomnia' OR 'Secondary Insomnia' OR 'sleep initiation and maintenance disorder' OR 'sleep initiation and maintenance disorders' OR 'Sleep Initiation Dysfunction' OR 'Sleep Initiation Dysfunctions' OR 'sleep initiation maintenance disorder' OR 'Sleep Qualities' OR 'Sleep Quality' OR 'sleeplessness' OR 'Transient Insomnia'):ab,ti,kw	37621
7	('randomised controlled study' OR 'randomised controlled trial' OR 'randomized controlled study' OR 'randomized controlled trial' OR 'random*' OR 'trial, randomized controlled' OR 'Clinical Trials, Randomized' OR 'Trials, Randomized Clinical'):ab,ti,kw	1375328
8	(#1 OR #2 OR #3) AND (#4 OR #5 OR #6) AND #7	7175

Table S4. The search strategy (WOS)

Search number	Query	Results
1	TS=((Angst) OR (Anxiety) OR (Anxiety Disorder*) OR (Anxiety Neuroses) OR (Anxiousness) OR (Hypervigilance) OR (Nervousness) OR (Neurotic Anxiety State*) OR (Social Anxieties) OR (Social Anxiety))	431889
2	TS=((agrypnia) OR (Chronic Insomnia) OR (DIMS) OR (disorder of sleep initiation and maintenance) OR (Disorders of Initiating and Maintaining Sleep) OR (disorders of sleep initiation and maintenance) OR (Early Awakening) OR (hyposomnia) OR (hyposomnias) OR (Insomnia Disorder*) OR (Insomnia*) OR (Nonorganic Insomnia) OR (Primary Insomnia) OR (Psychophysiological Insomnia) OR (Rebound Insomnia) OR (Secondary Insomnia) OR (sleep initiation and maintenance disorder) OR (sleep initiation and maintenance disorders) OR (Sleep Initiation Dysfunction) OR (Sleep Initiation Dysfunctions) OR (sleep initiation/maintenance disorder) OR (Sleep Qualities) OR (Sleep Quality) OR (sleeplessness) OR (Transient Insomnia))	138739
3	TS=((randomised controlled study) OR (randomised controlled trial) OR (randomized controlled study) OR (randomized controlled trial) OR (random*) OR (trial, randomized controlled) OR (Clinical Trials, Randomized) OR (Trials, Randomized Clinical))	2637729
4	#3 AND #2 AND #1	4594

Table S5. The search strategy (CNKI)

Search number	Query	Results
1	焦虑 ＋ 焦虑症	149735
2	睡眠障碍 + 失眠 + 不寐 + PQSI + ISI	56203
3	抑郁	140802
4	#1 AND #2 NOT #3	2308

Table S6. The search strategy (WF)

Search number	Query	Results
1	焦虑 OR 焦虑症	342039
2	睡眠障碍 OR 失眠 OR 不寐 OR PQSI OR ISI	119390
3	抑郁	407489
4	#1 AND #2 NOT #3	5277

Table S7. The search strategy (VIP)

Search number	Query	Results
1	焦虑 OR 焦虑症	87081
2	睡眠障碍 OR 失眠 OR 不寐 OR PQSI OR ISI	47909
3	抑郁	11654
4	#1 AND #2 NOT #3	1146

Table S8. The search strategy (Sinomed)

Search number	Query	Results
1	焦虑 OR 焦虑症	342039
2	睡眠障碍 OR 失眠 OR 不寐 OR PQSI OR ISI	119390
3	抑郁	407489
4	#1 AND #2 NOT #3	4003
