# Supplementary figures and images for: Efficacy of non-pharmacological interventions for alleviating insomnia in individuals with generalized anxiety disorder: systematic evaluation and net meta-analysis
Source: Front Psychiatry. 2025 Oct 31;16:1669888. doi: 10.3389/fpsyt.2025.1669888 (PMC12615457; doi:10.3389/fpsyt.2025.1669888)

A

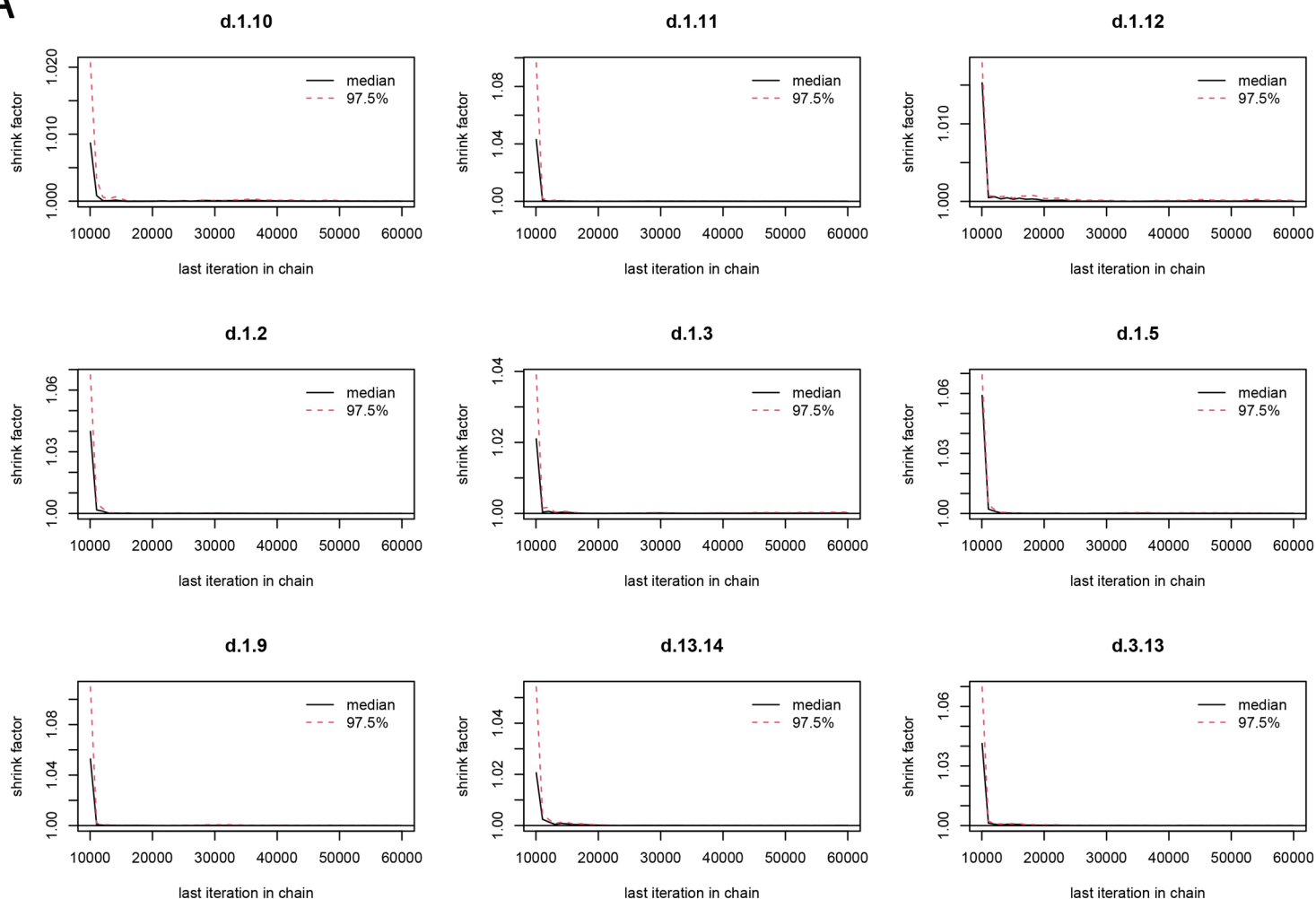

B

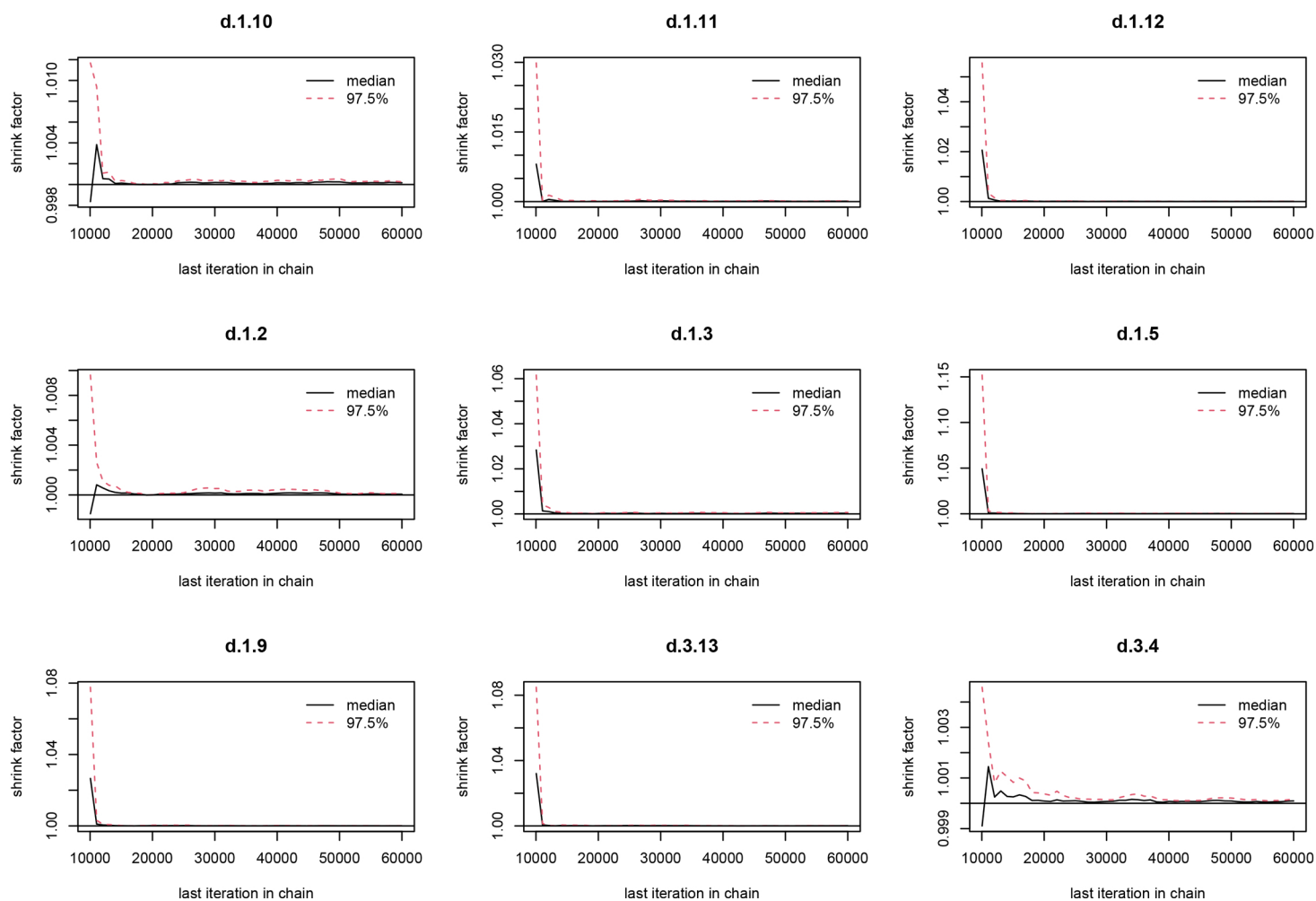

Supplement: Supplementary File S4 — Potential Scale Reduction Factor (PSRF) Plots for Convergence Diagnosis (A) Sleep quality outcome (B) Anxiety outcome. [file SupplementaryFile4.pdf]

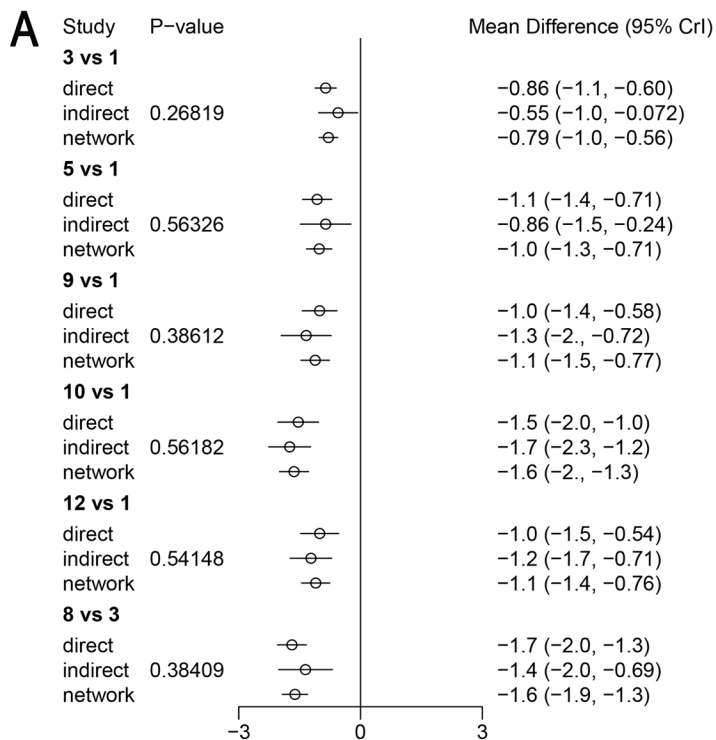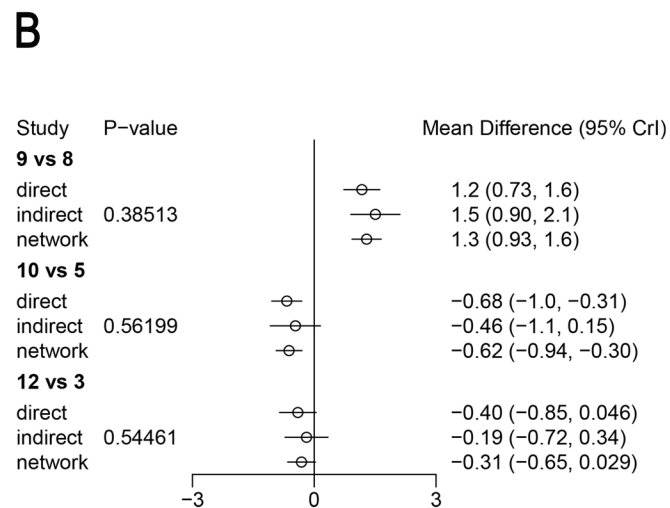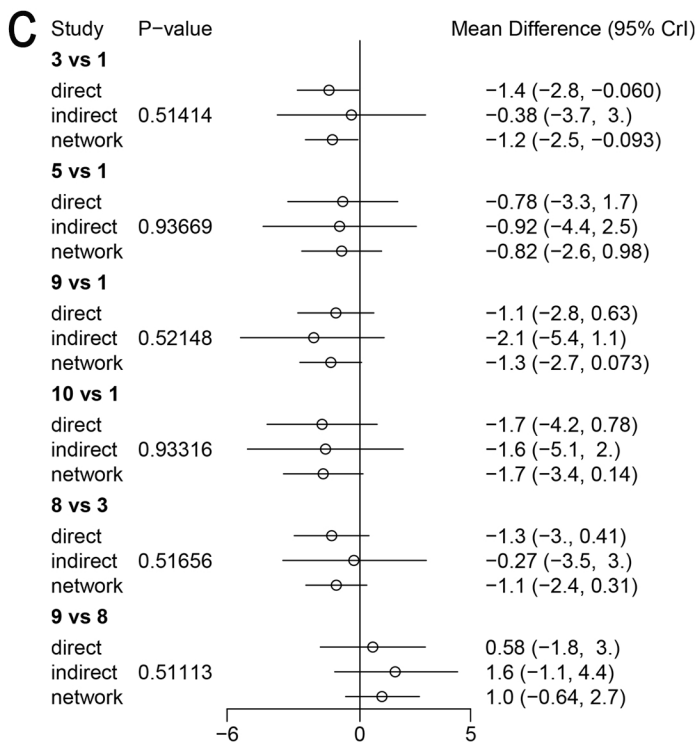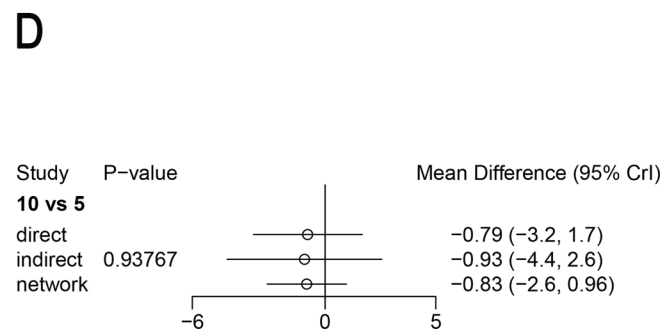

Supplement: Supplementary File S5 — Node-Splitting Analysis for Local Inconsistency Assessment (A, B) Sleep quality outcome (C, D) Anxiety outcome. [file SupplementaryFile5.pdf]
